# The Functional Connectivity Between the Nucleus Accumbens and the Ventromedial Prefrontal Cortex as an Endophenotype for Bipolar Disorder

**DOI:** 10.1016/j.biopsych.2018.07.023

**Published:** 2018-12-01

**Authors:** Joseph R. Whittaker, Sonya F. Foley, Edward Ackling, Kevin Murphy, Xavier Caseras

**Affiliations:** a Cardiff University Brain Research Imaging Centre - CUBRIC, Cardiff, United Kingdom; b School of Physics and Astronomy at Cardiff University, Cardiff, United Kingdom; c MRC Centre for Neuropsychiatric Genetics and Genomics at Cardiff University, Cardiff, United Kingdom; d Division of Psychological Medicine and Clinical Neurosciences at Cardiff University, Cardiff, United Kingdom

**Keywords:** Bipolar disorder, Connectivity, fMRI, Nucleus accumbens, Psychosis, Ventromedial prefrontal cortex

## Abstract

**Background:**

Alterations in functional connectivity between the nucleus accumbens (NAcc) and frontal cortices have been previously associated with the presence of psychiatric syndromes, including bipolar disorder (BD). Whether these alterations are a consequence or a risk factor for mental disorders remains unresolved.

**Methods:**

This study included 35 patients with BD, 30 nonaffected siblings of patients with BD, and 23 healthy control subjects to probe functional connectivity at rest between NAcc and the rest of the brain in a cross-sectional design. Blood oxygen level–dependent time series at rest from NAcc were used as seed region in a voxelwise correlational analysis. The strength of the correlations found was compared across groups after Fisher’s *Z* transformation.

**Results:**

We found increased functional connectivity between the NAcc and the ventromedial prefrontal cortex—comprising mainly the subgenual anterior cingulate—in patients compared with healthy control subjects. Participants at increased genetic risk but yet resilient—nonaffected siblings—showed functional connectivity values midway between the former two groups.

**Conclusions:**

Our results are indicative of the potential for the connectivity between NAcc and the ventromedial prefrontal cortex to represent an endophenotype for BD.

Psychiatric disorders have a profound personal impact on patients and their families and carry an extraordinary societal burden (1). Despite increasing research efforts in the field, little advance has been made with regard to our mechanistic understanding of psychiatric disorders, limiting our ability to develop new efficient interventions (2). Recent neuroimaging research has focused on investigating the functional networks of the brain, under the assumption that psychopathology arises from alterations in connectivity/wiring of the brain 2, 3. From a functional magnetic resonance imaging (fMRI) perspective, recently accumulated evidence suggests the presence of altered brain connectivity, i.e., coactivation of different brain structures required to work together to efficiently produce a complex response, in most severe psychiatric disorders [e.g. 4, 5, 6] and among the functional networks the so-called reward network (7).

The reward network has been described as a corticobasal ganglia circuit, in which the ventral striatum—mainly the nucleus accumbens (NAcc) in humans—occupies a central role, despite including a large number of subcortical and cortical regions [see (8) for a comprehensive description of this network]. This network underpins reward seeking and hedonic responses to positive stimuli, which are basic to animal and human behavior (9). Within this network, interaction of the NAcc with the ventromedial prefrontal cortex (vmPFC) has been shown to be pivotal in regulating responses to reward and emotional symptoms in psychiatric disorders 10, 11, 12. For example, previous research has stressed the relevance of abnormal NAcc activation in bipolar disorder (BD) 13, 14, 15, 16 and of its interaction with the vmPFC (17) during reward anticipation and receipt.

The present study aimed to examine the existence of NAcc functional connectivity alterations in BD during the absence of acute symptoms. We hypothesized that patients with BD recruited during euthymia would present alterations in NAcc functional connectivity compared with low-risk healthy control (HC) subjects. We expected these potential alterations to include parts of the vmPFC. The fact that our participants would be euthymic at the time of scanning would indicate that any alterations detected would represent a trait of the disorder, rather than a reflection of a psychopathological state. We were also interested in examining whether any alterations detected would extend into an at-risk but yet resilient sample of participants—nonaffected siblings of patients with BD older than the most frequent age of onset for the disorder. Under the assumption of functional connectivity alterations representing an endophenotype (18) of BD, we hypothesized nonaffected sibling (SIB) participants to fall between patients with BD and HC subjects in terms of the strength of any connectivity alterations detected. Owing to existing discrepancies with regard to the potential direction of these effects (4), we did not make any a priori assumptions as to whether functional connectivity would appear increased or decreased in patients with BD and SIB participants relative to HC subjects.

## Methods and Materials

### Participants

One hundred participants were recruited for this study (43 patients with BD, 34 SIB participants, and 23 HC subjects). Participants with a previous diagnosis of BD were recruited through the National Centre for Mental Health after confirmation of their diagnosis by a trained clinician (XC) using the Mini-International Neuropsychiatric Interview (19). The Mini-International Neuropsychiatric Interview was also used in SIB participants and HC subjects to ascertain for potential psychopathology. SIB participants were contacted through recruited patients with BD, and only one sibling per family was included. HC subjects were recruited from the community via advertisement. General inclusion/exclusion criteria for all groups included 1) age 35 to 60 years; 2) no personal history of psychotic or borderline personality disorder; 3) no recent history (within the past year) of abuse of, or dependence on, alcohol or other substances; and 4) conforming to Cardiff University Brain Research Imaging Centre standard MRI safety protocols. Specific criteria for patients with BD included 1) positive diagnosis of BD type I or type II; 2) mood stability, defined as absence of major mood episodes and no changes in medication for at least 1 month before scanning; and 3) current euthymia, defined as current scores below 10 in both the Hamilton Depression Rating Scale (HDRS) (20) and the Young Mania Rating Scale (YMRS) (21). In the case of SIB participants, an added criterion was no personal history of any mood or psychotic episode. For HC subjects, additional criteria were 1) no personal history of any mental disorder and 2) no family history in first-degree relatives of any mood or psychotic disorders. In addition to the above-mentioned clinical instruments, participants completed the National Adult Reading Test (22) to estimate premorbid IQ.

Three participants decided to withdraw from the MRI scan, 2 no longer met inclusion/exclusion criteria on the day of the scan, and 7 were excluded owing to failing data quality assurance (see Data Analysis). Therefore, the final sample included 35 patients with BD, 30 SIB participants, and 23 HC subjects. All participants gave written informed consent after receiving a complete description of the study and were paid £20 for participating. The study was approved by the local National Health Service Research Ethics Committee.

### Imaging Protocol

Resting-state fMRI data were acquired with a gradient-recalled echo echo-planar imaging (EPI) sequence (repetition time = 2.5 seconds; echo time = 35 ms; flip angle = 90°; field of view = 211 mm; 3 mm^2^ in-plane resolution; Array Spatial Sensitivity Encoding Technique acceleration factor = 2) using a Signa HDx 3.0T MRI scanner (GE Healthcare, Chicago, IL) equipped with an eight-channel receiver head coil. The scan duration was 7.5 minutes, during which participants were instructed to lie still and keep their eyes closed, and foam cushions were used inside the head coil to minimize the degree of motion. Whole-brain coverage was achieved with 44 interleaved slices (3-mm thickness, 0.3-mm gap). A total of 180 volumes were acquired, plus 4 dummy volumes to allow the longitudinal magnetization to reach a steady state. To register resting-state scans to standard space, a three-dimensional fast spoiled gradient echo image was also acquired (repetition time = 7.9 seconds; echo time = 3 ms; inversion time = 450 ms; flip angle = 20°; 1 mm^3^ isotropic resolution).

### Data Analysis

#### Preprocessing

Data were first preprocessed and registered to a standard space using a pipeline constructed from the AFNI (http://afni.nimh.nih.gov/afni) and FSL (https://fsl.fmrib.ox.ac.uk/fsl/fslwiki) software packages. The preprocessing pipeline was designed to mitigate the effect of subject motion and physiological noise as far as possible and consisted of the following steps: 1) despiking (3dDespike; AFNI); 2) motion correction (3dvolreg; AFNI) by registering all volumes to the first one; 3) nuisance regression (3dREMLfit; AFNI) with prewhitening (23) to remove noise related to cardiac and respiratory systems 24, 25, 26 and variance related to residual motion—only the six estimated motion parameters, and not their temporal derivatives, were used to model residual motion variance so as to do so parsimoniously and thus limit the potential for removing variance of interest (27); 4) slice time correction (3dTshift; AFNI); 5) nonlinear registration to 2 mm Montreal Neurological Institute template (FLIRT and FNIRT; FSL); 6) ANATICOR (28), bandpass filtering (0.01–0.8 Hz), and motion scrubbing performed in a single step (3dTproject; AFNI). ANATICOR regressors were derived from the lateral ventricles and a global white matter mask (FAST; FSL) as well as voxelwise local white matter regressors to minimize sensitivity to motion (28). Volumes considerably corrupted by motion (and scrubbed) were defined as volumes with a framewise displacement (Euclidean norm) greater than 0.25 mm, and subjects with greater than 20% of volumes corrupted were excluded from further analysis. This threshold ensured that all included subjects had the equivalent of at least 6 minutes of resting fMRI data (Supplemental Figure S1 presents a post hoc comprehensive analysis of movement, showing that this did not differ across groups and was not a confounding factor for our main results).

#### Seed-Based Functional Connectivity Analysis

For each subject, an NAcc seed time series was derived from the bilateral nucleus accumbens probability mask included in the Harvard-Oxford subcortical atlas. From this NAcc mask, a seed time series was created by taking a probability-weighted mean, ensuring that the contribution of individual voxel time series was determined by their probability of being part of this NAcc-based mask. For each subject, the correlation between each voxel in the brain and the seed time course was calculated (3dTcorr1D; AFNI), and the resulting statistical parameter maps were spatially smoothed by 8 mm (full width at half maximum) (3dmerge; AFNI) and Fisher’s *Z* transformed.

**Figure 1:**
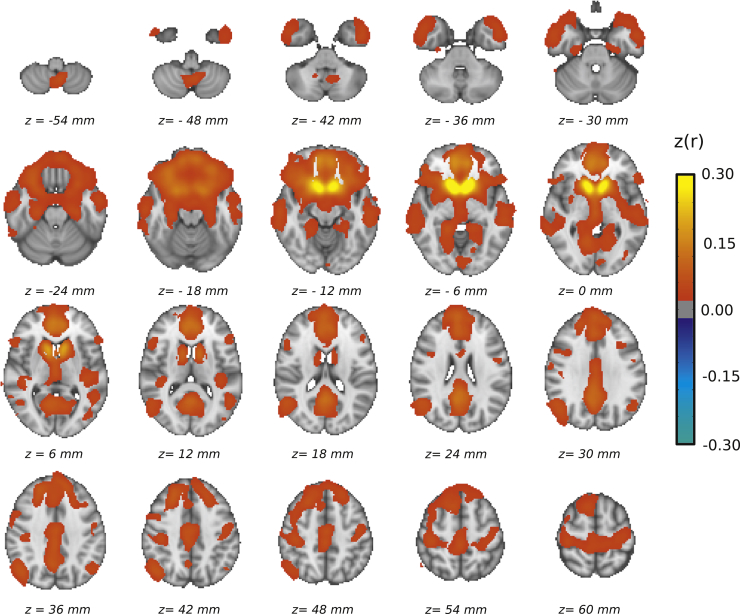
Ventral striatum brain network obtained from the whole sample. Warm-colored areas showed positive correlation (corrected *p* < .05) with the average blood oxygen level–dependent time course in the nucleus accumbens (yellow) obtained from the Harvard-Oxford subcortical atlas. No negative correlation survived correction for multiple comparisons. Color indicates size of the association (Fisher *Z*-transformed correlation coefficient) according to the provided scale.

The NAcc functional connectivity network (Figure 1) was derived from all participants using a one-sample *t* test (3dttest++; AFNI). Voxelwise differences between groups were tested for using a two-sample *t* test (-clustsim option in 3dttest++; AFNI). To account for multiple tests, a model-free nonparametric approach was used (3dttest++). This permutation approach does not use a mathematical model for the spatial autocorrelation function and is the most robust method for keeping false-positive rates at the nominal level for simple statistical models (29). Voxels included in the comparisons were confined to a mask derived from mean EPI across all subjects, thus excluding areas of substantial signal dropout, discounting voxels from the lateral ventricles and white matter–eroded masks taken from Harvard-Oxford atlas.

Post hoc tests at the region of interest level were performed to examine group differences in SIB participants versus HC subjects and patients with BD versus SIB participants in the vmPFC region of interest that was identified in the voxelwise patients with BD versus HC subjects comparison. The mean *Z*-transformed correlation coefficient for the significant cluster was calculated for each participant and compared between SIB participants and HC subjects by means of Welch’s *t* test and between SIB participants and patients with BD by means of paired *t* test.

## Results

### Group Characterization

Groups did not differ in age (*F*_2,87_ = 0.85, *p* > .1), gender distribution (χ^2^_2_ = 0.06, *n* = 88, *p* > .1), performance on the National Adult Reading Test (*F*_2,76_ = 0.59, *p* > .1), or weekly intake of alcohol units (*F*_2,81_ = 1.12, *p* > .1). As expected, groups differed in clinical symptoms, with patients with BD showing higher scores in the YMRS (*F*_2,85_ = 13.43, *p* < .001) and HDRS (*F*_2,85_ = 18.47, *p* < .001) than HC subjects and SIB participants, albeit still indicative of low presence of mood symptoms in accordance with their euthymic state (Table 1).

**Table 1:** Demographics and Clinical Symptoms for Participants Included in Connectivity Analyses

	HC Subjects (*n* = 23)	Patients With BD (*n* = 35)	SIB Participants (*n* = 30)
Gender, Male, *n* (%)	9 (39%)	13 (37%)	12 (40%)
Age, Years	44.00 (4.48)	44.71 (5.51)	46.03 (6.94)
NART	38.11 (4.47)	36.12 (6.89)	36.65 (6.12)
EtOH	7.02 (8.36)	12.79 (16.67)	11.96 (14.27)
YMRS	0.26 (0.61)	3.31 (3.87)	0.66 (0.84)^a^
HDRS	0.35 (0.88)	3.97 (3.78)	0.90 (1.12)^a^

All values except gender are presented as mean (SD). NART score was missing for 11 participants (owing to time constrains or English not being their first language), EtOH weekly intake data were missing for 6 participants, and YMRS and HDRS scores were missing for 2 participants.

BD, bipolar disorder; EtOH, ethyl alcohol consumption (in weekly units); HC, healthy control; HDRS, Hamilton Depression Rating Scale; NART, National Adult Reading Test (number of correct answers is reported); SIB, nonaffected sibling; YMRS, Young Mania Rating Scale.

a BD > HC, SIB.

Patients with BD in our sample (16 with BD type I and 19 with BD type II) experienced their first significant mood episode on average at 18 years of age but received a diagnosis of BD 12 years later on average. This delay in diagnosis could be partly explained by the fact that greater than 90% of these patients experienced low mood as their first episode and were initially treated for depression. Six (17%) patients with BD presented with current comorbid anxiety disorders—agoraphobia being the most prevalent—but that figure rose to over a third of the sample (37%) when considering a lifetime prevalence. Owing to the strict inclusion criteria, no other diagnoses were present in this sample. At the time of scanning, only 4 patients with BD were not taking psychotropic medication, over a third were taking antidepressants (*n* = 14) and/or antipsychotics (*n* = 13), and over half were taking mood stabilizers (*n* = 21). Half of the patients with BD (*n* = 17) were prescribed a combination of at least two of the above classes of drugs. Only 2 SIB participants presented with a mental health history—past history of generalized anxiety disorder (*n* = 1) and health anxiety (*n* = 1). Following our inclusion criteria, HC subjects had no history of any psychiatric disorder; and neither HC subjects nor SIB participants were taking any psychotropic medication at the time of this study.

### Blood Oxygen Level–Dependent Times Series in the Ventral Striatum Correlates With vmPFC in the Overall Sample

To capture the entire NAcc connectivity network, we ran a voxelwise correlation analysis using the blood oxygen level–dependent (BOLD) time series in the NAcc as reference. Several brain areas were shown to be positively associated with the NAcc BOLD time course across the whole sample (Figure 1). These included subcortical structures bilaterally, such as the caudate, amygdala, hippocampus, parahippocampal gyri, ventral thalamus, and anterior putamen, and cortical areas, such as the vmPFC, ventrolateral prefrontal cortex, anterior cingulate, superior frontal gyrus, middle temporal gyrus, lingual gyrus, and postcentral gyrus. No negative correlations survived correction for multiple comparisons.

### Patients With BD and HC Subjects Differ in Connectivity Between Ventral Striatum and vmPFC

**Figure 2:**
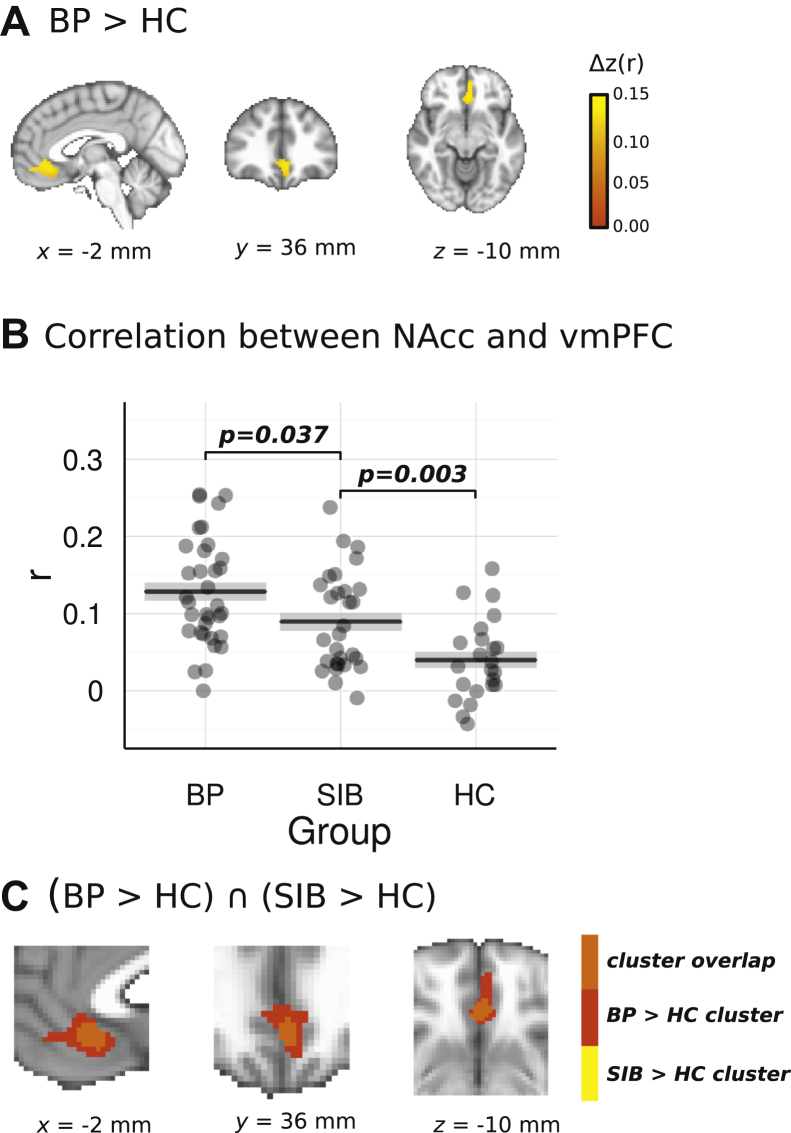
**(A)** Cluster in the ventromedial prefrontal cortex (vmPFC) showing increased association with the average blood oxygen level–dependent time course in the ventral striatum in patients with bipolar disorder (BD) compared with healthy control (HC) subjects (voxelwise whole-brain comparison). Color scale indicates the size of the difference (*Z*-transform) between BD and HC. **(B)** Intensity of the association between the average blood oxygen level–dependent time course from the ventral striatum seed region and the vmPFC cluster obtained from the voxelwise patients with BD vs. HC subjects comparison for each experimental group. *p* values correspond to Welch’s *t* tests of the *Z*-transformed Pearson correlation between nonaffected sibling (SIB) participants and each of the other two groups. **(C)** Overlap between the cluster in panel **(A)** and the equivalent cluster obtained in the voxelwise exploratory (uncorrected *p* < .01) comparison between SIB participants and HC subjects. NAcc, nucleus accumbens.

Based on the whole-brain correlation map shown in Figure 1, the voxelwise comparison of patients with BP versus HC subjects resulted in a significant cluster in the vmPFC (center of mass at Montreal Neurological Institute *x* = −2, *y* = 36, *z* = −10, 334 voxels, cluster-extent corrected *p* < .05), predominantly left lateralized (Figure 2A). This indicated an increased positive association between BOLD time course in the NAcc and the vmPFC in patients with BD compared with HC subjects, but with this correlation within the two groups being significantly nonzero (*p* < .001). In light of recent research showing antipsychotic medication affecting resting-state corticostriatal connectivity (30), we compared the *Z*-transformed NAcc-vmPFC correlation between patients receiving this class of medication and patients not taking antipsychotics, resulting in no significant effects (*t*_33_ = 0.97, *p* > .1); there was also no significant association—albeit there was a trend—between chlorpromazine equivalents of antipsychotic dosage and NAcc-vmPFC correlation within the BD group (*r* = .30, *n* = 35, *p* = .08). Lithium dosage (*r* = −.38, *n* = 9, *p* > .1) or presence of mood stabilizers, i.e., lithium or antiepileptics (*t*_33_ = 0.06, *p* > .1), did not significantly predict NAcc-vmPFC correlation.

**Figure 3:**
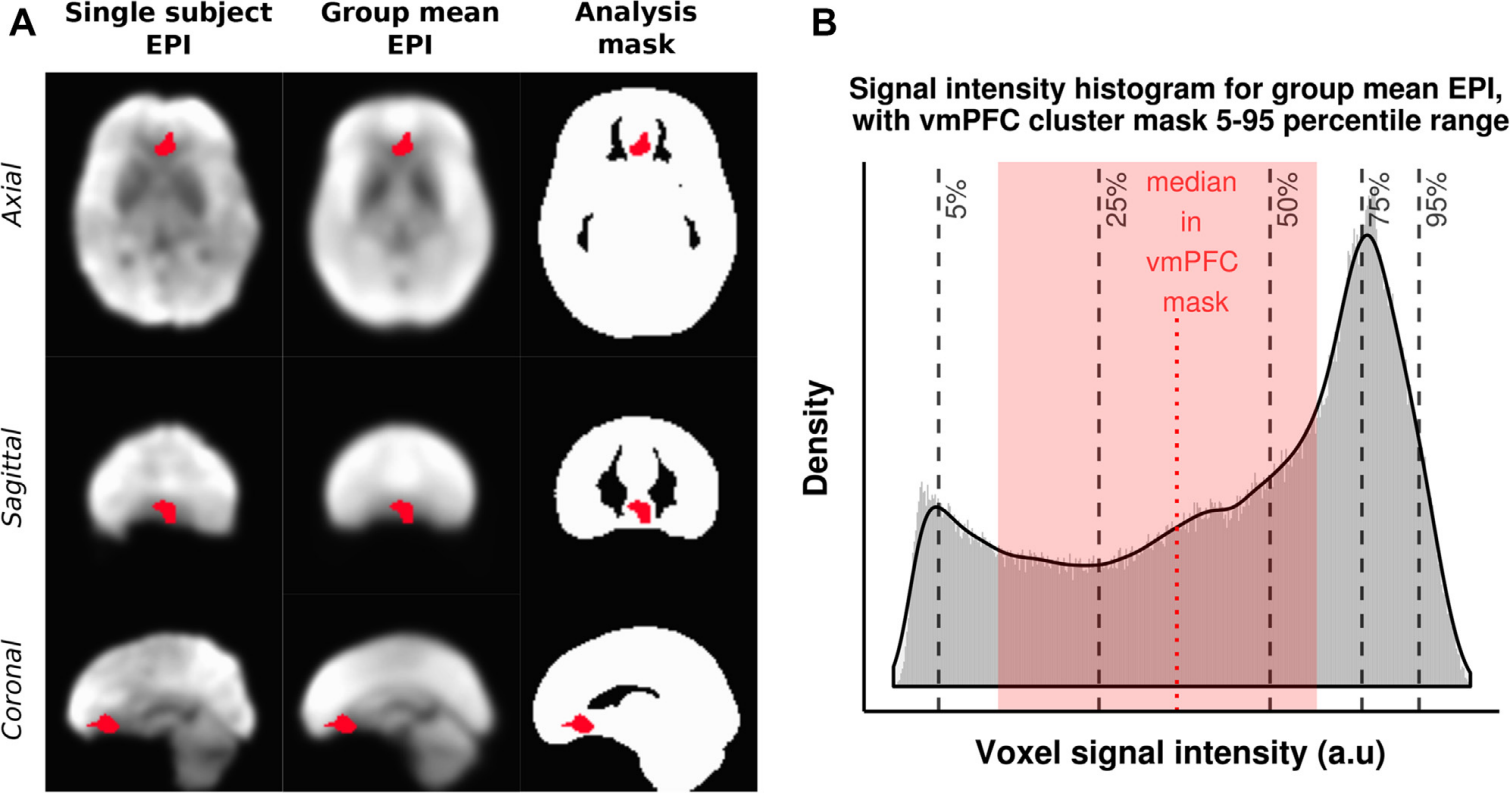
**(A)** Column 1 shows the mean echo-planar imaging (EPI) image from a representative participant, column 2 shows the group mean EPI image, and column 3 shows the analysis mask. In each case, the ventromedial prefrontal cortex (vmPFC) cluster mask is overlaid. **(B)** Histogram of voxel intensity values from the mean EPI image across all participants, with 5th, 25th, 50th, 75th, and 95th percentiles marked. The median signal intensity from the vmPFC is marked, and the 5th–95th percentile range is shown. a.u., arbitrary units.

Owing to its proximity to the frontal sinuses, the vmPFC is an area of the brain that is susceptible to signal dropout during EPI acquisitions. By restricting our analysis to a mask derived from EPI images themselves (Figure 3A), we ensured that only voxels with sufficient signal were included. Figure 3B includes a histogram of the signal intensity from the group mean EPI data, showing that the 5th to 95th percentile range of voxels in the vmPFC cluster fall comfortably in the robust range of voxels, indicating that signal dropout did not significantly degrade voxels in the vmPFC cluster.

### SIB Participants Show Lower Ventral Striatum–vmPFC Than Patients With BD, but Higher Ventral Striatum–vmPFC Than HC Subjects

Post hoc region of interest analyses using the vmPFC mask from the above analysis to compare NAcc-vmPFC connectivity between SIB participants and HC subjects and SIB participants and patients with BD resulted in a significant group difference for both the former (*t*_49.93_ = 3.09, *p* < .005) and the latter (*t*-paired_23_ = 2.21, *p* < .05) comparisons (Figure 2B), with SIB participants showing an average correlation midway between the BD and the HC average correlations. To validate these results, we performed exploratory voxelwise group comparisons (uncorrected *p* < .01) using SIB participants as the reference group. For SIB > HC, a significant cluster emerged in the vmPFC with a center of mass at Montreal Neurological Institute *x* = −2, *y* = 32, *z* = −10 containing 95 voxels, of which 94 overlapped with the BD > HC cluster (Figure 2C). No clusters emerged in the vmPFC when comparing SIB and BD groups.

### NAcc-vmPFC Connectivity Association With Residual Symptoms

The strength of NAcc-vmPFC connectivity, i.e., *Z*-transform correlation index, did not appear to be associated with the presence of residual symptoms of depression (HDRS score, *r* = −.02, *p* > .1) or mania (YMRS score, *r* = −.18, *p* > .1) in our BD sample. Despite the lack of association, we added HDRS and YMRS scores as covariates in our group comparisons to ascertain for any potential confounding effects. As expected, results remained the same as previously reported.

## Discussion

The main aim of this study was to investigate potential alterations in functional connectivity between the NAcc and vmPFC in patients with BD and to examine whether similar alterations could be found in unaffected siblings of patients with BD. Our results show these two brain regions to be positively associated within all groups but differing in intensity across patients with BD, SIB participants, and unaffected unrelated HC subjects, suggesting that this biomarker may behave as an endophenotype for this disorder.

Our whole-brain correlational analysis using the NAcc as seed region showed an extensive associative network that largely overlapped with previous published research 31, 32, 33. Importantly, a whole-brain comparison between BD and HC showed that these two groups differed only in the intensity of the association between NAcc and vmPFC—the latter mainly consisting of the left pregenual anterior cingulate and the medial orbitofrontal cortex—with patients with BD presenting increased connectivity compared with HC. Ventral frontostriatal hyperconnectivity had been previously reported in both patients with psychosis and participants at risk for psychosis, and was suggested to be indicative of an excessive motivational drive that could give rise to psychotic experiences (34). However, excessive metabolic activity within and connectivity from this cortical area have also been associated with depression (35), and this area has been successfully used as a deep brain stimulation target for treatment-resistant depression 36, 37 and BD (38). Recent animal research shows that activation of vmPFC induces inhibition of midbrain dopaminergic interactions with the ventral striatum causing suppression of reward-seeking behavior and therefore increased depressive-like symptoms in rats (12). Our results, though, suggest that abnormal connectivity between vmPFC and NAcc in humans could represent a trait marker of mental disorder rather than merely indicating the presence of depressive symptoms, as, first, our patients with BD were scanned during euthymia, and, second, the strength of this connectivity did not appear to correlate with the presence of residual depressive or manic symptoms in our patients with BD.

Importantly, differences in connectivity between the NAcc and the vmPFC cluster were also found in SIB participants compared with HC subjects. It is worth noting that our SIB sample represents a resilient group of genetically high-risk participants. BD is highly heritable, and first-degree relatives of patients carry an excess risk of the disorder (39). However, our SIB participants were recruited solely based on absence of any personal history of mood disorders and psychosis and age older than 35 years, indicating lower probability of developing BD or psychosis in the future (40). As such and considering also that consequently none of our SIB participants were taking psychotropic medication, alterations in the connectivity between these two brain areas can be considered a premorbid risk marker for BD and a potential endophenotype (41) for the disorder.

Our study has some limitations. As with most research including patients with a chronic disorder, a potential confounding effect of medication is present. However, a recent review suggests that the effects of medication have less of an impact on brain function than originally thought, and where brain function is affected, this could mainly be in the form of type II errors owing to normalization (42). Furthermore, any medication effect would affect only the results from patients with BD, and we have shown antipsychotics not to be associated with our main finding in this group. Moreover, the fact that we replicate the same finding—albeit attenuated—in nonmedicated SIB participants leads us to believe that our results are not a consequence of medication status. Also, owing to the modest sample size and to avoid the need for more stringent corrections for multiple comparisons, we could not further exploit the data to investigate, for example, potential compensatory mechanisms present in SIB participants that could explain resilience or potential BD subtype differences with regard to NAcc-vmPFC connectivity. Future studies designed to answer these questions are warranted. Finally, the lack of any behavioral output related to reward seeking or hedonic reaction to reward in our study precludes the possibility to validate the hypothesis that the altered connectivity found in this study relates to reward processing, although previous preclinical (43) and clinical (44) research would suggest that it is.

In summary, our results show an increased connectivity during rest between NAcc and vmPFC in euthymic patients with BD compared with HC subjects. SIB participants also showed increased connectivity between these brain areas compared with HC subjects, albeit attenuated compared with affected siblings of patients. Therefore, we propose that this connectivity alteration represents a premorbid biomarker for BD with high potential to represent an endophenotype for this disorder associated with reward function. Future similar research in unipolar depression, schizophrenia, or addiction, for which alterations in reward function have been described, should determine whether this biomarker is common to other mental disorders.
