# Supplementary material for: The Functional Connectivity Between the Nucleus Accumbens and the Ventromedial Prefrontal Cortex as an Endophenotype for Bipolar Disorder
Source: Biol Psychiatry. 2018 Dec 1;84(11):803–9. doi: 10.1016/j.biopsych.2018.07.023 (PMC6218647; doi:10.1016/j.biopsych.2018.07.023)
Supplement: Supplemental Figure [file mmc1.pdf]

The Functional Connectivity Between Nucleus Accumbens and the Ventromedial Prefrontal Cortex as an Endophenotype for Bipolar Disorder

Supplemental Information

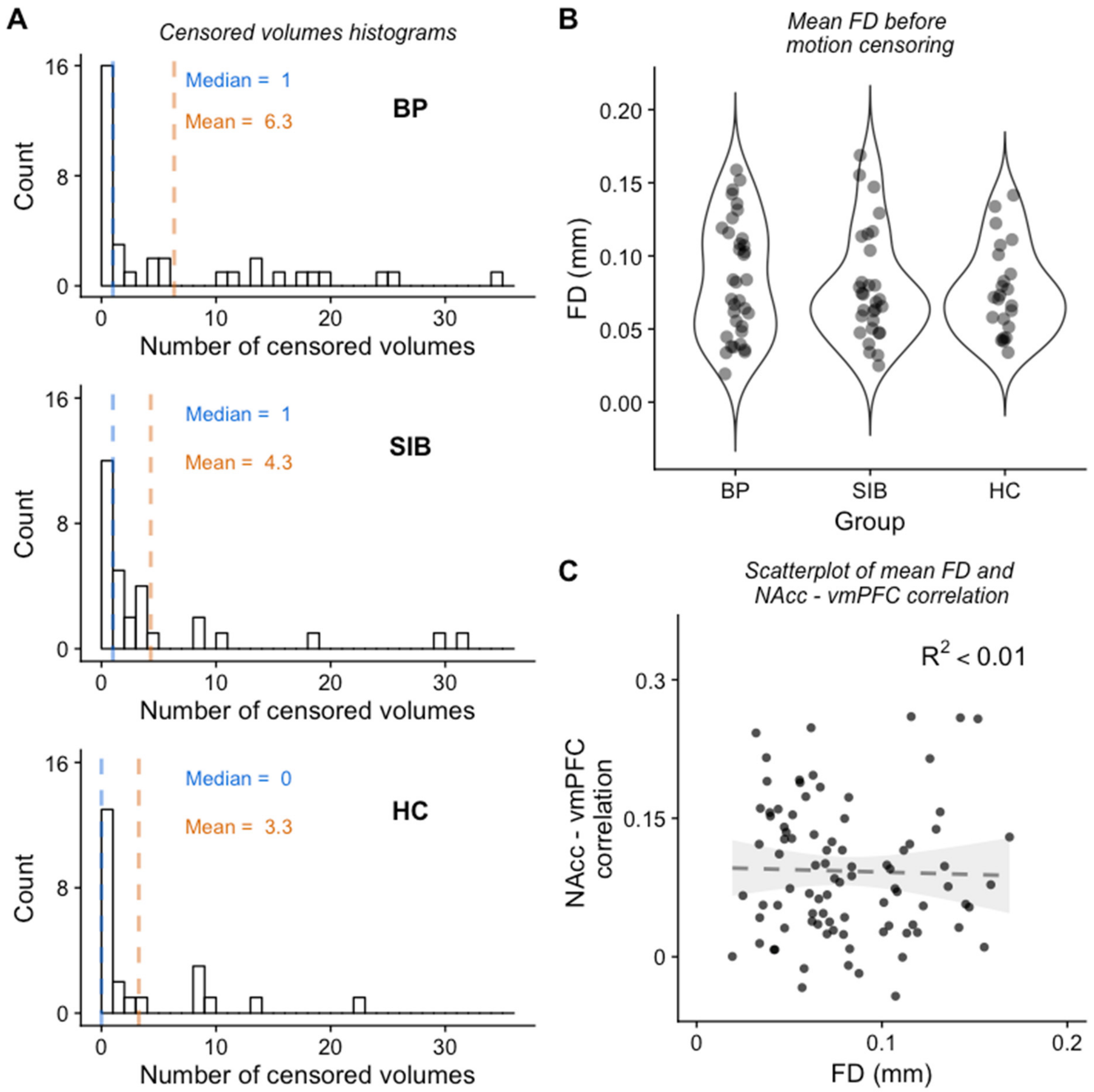

Supplementary Figure S1. A) Distribution of the number of volumes censored for each group; in all cases the median was low and for each group the majority of participants had no censored volumes. B) Participant's mean FD values across groups, for which there were no significant differences. C) Scatter plot of mean FD plotted against the NAcc-vmPFC correlation. It can be concluded that participant's motion did not contribute to the NAcc-vmPFC association.
